# Bioactive Molecules from the Innate Immunity of Ascidians and Innovative Methods of Drug Discovery: A Computational Approach Based on Artificial Intelligence

**DOI:** 10.3390/md22010006

**Published:** 2023-12-20

**Authors:** Laura La Paglia, Mirella Vazzana, Manuela Mauro, Alfonso Urso, Vincenzo Arizza, Aiti Vizzini

**Affiliations:** 1 Istituto di Calcolo e Reti ad Alte Prestazioni–Consiglio Nazionale delle Ricerche, Via Ugo La Malfa 153, 90146 Palermo, Italy; laura.lapaglia@icar.cnr.it (L.L.P.); alfonso.urso@icar.cnr.it (A.U.); 2 Dipartimento di Scienze e Tecnologie Biologiche, Chimiche e Farmaceutiche–Università di Palermo, Via Archirafi 18, 90100 Palermo, Italy; mirella.vazzana@unipa.it (M.V.); manuela.mauro01@unipa.it (M.M.); vincenzo.arizza@unipa.it (V.A.)

**Keywords:** bioactive molecules, marine invertebrate, artificial intelligence, innate immunity

## Abstract

The study of bioactive molecules of marine origin has created an important bridge between biological knowledge and its applications in biotechnology and biomedicine. Current studies in different research fields, such as biomedicine, aim to discover marine molecules characterized by biological activities that can be used to produce potential drugs for human use. In recent decades, increasing attention has been paid to a particular group of marine invertebrates, the Ascidians, as they are a source of bioactive products. We describe omics data and computational methods relevant to identifying the mechanisms and processes of innate immunity underlying the biosynthesis of bioactive molecules, focusing on innovative computational approaches based on Artificial Intelligence. Since there is increasing attention on finding new solutions for a sustainable supply of bioactive compounds, we propose that a possible improvement in the biodiscovery pipeline might also come from the study and utilization of marine invertebrates’ innate immunity.

## 1. Introduction

Ascidians are tunicates, marine invertebrate chordates, considered the sister group of vertebrates [1,2,3,4]. They owe their name to the tunic, an epidermally secreted external layer that envelops the body. The tunic is composed of an ECM rich in collagen and tunicin (a form of cellulose) and also rich in immune cells [5,6,7,8,9]. The tunic also contains proteins with 3,4-dihydroxyphenylalanine (DOPA), with a catechol moiety involved in the first line of immune reaction [10] and wound healing [11,12], and 3,4,5-trihydroxyphenylalanine (TOPA) with a pyrogallol moiety [13]. Ascidians are the source of many bioactive molecules belonging to a wide variety of chemical categories [14,15] and with potential health applications, including cytotoxic, antimitotic, antiviral and antimicrobial compounds [16,17,18,19]. Most of the metabolites synthesized by ascidians contribute to creating the physico-chemical barrier preventing the entrance of foreign organisms into the internal fluids or the colonization of the tunic by encrusting organisms. The most represented chemical classes among the bioactive secondary metabolites isolated from tunicates are alkaloids, polyketides, and peptides [18]. Cytotoxicity against mammalian cell lines and anti-proliferative activity were the most frequently assigned bioactivities. Compounds with cytotoxic and antineoplastic properties isolated from ascidians belong to disparate chemical classes, and three of them have entered clinical trials [20]. Due to their key phylogenetic position in chordate evolution, the ascidians are a powerful model for studying innate immunity [4]. They possess an exclusively innate immune system, including inflammatory, humoral, and cellular responses. On an evolutionary level, inflammation is a highly conserved phenomenon and appears to be an essential first line of defense for both invertebrates and vertebrates. The innate immune system is the major contributor to acute inflammation [21,22], a rapid protective response to microbial infection, tissue injury, and insults [23], and the principal promoter of inflammatory responses often involves infection by microbial invaders or exposure to foreign particles/irritants/pollutants [24].

When host cells capable of innate immune responses encounter pathogenic microbes or other foreign or host irritants, the inflammatory response initiates within minutes. The host cells recognize the stimulus through various sensing mechanisms involving trans-membrane receptors. In *Ciona robusta* (previously *Ciona intestinalis*), these interactions transmit signals to the nucleus, resulting in the activation and regulation of numerous genes via both transcriptional and post-transcriptional mechanisms [21,25,26,27,28,29,30,31,32,33], such as antimicrobial peptides and complement factors [34,35] and proinflammatory cytokines and chemokines which activate endothelial cells and recruit immune system cells to the infection site [36,37,38].

The immune system is made up of a complex and dynamic network of cell subsets and mediators that promote host defense from infectious agents or tumor cells and maintain immunological tolerance in the organisms [39,40]. Vertebrate immunity is classically divided into innate and adaptive immune systems that act interdependently based on bidirectional crosstalk [41]. It is well known that the innate immune system provides the first line of defense in immune response and can induce and regulate many different adaptive immunity functions [42].

This review provides an overview of the bioactive molecules isolated from the innate immune system response of ascidians and, at the same time, describes a new approach combining omics technologies and new bioinformatic strategies for drug discovery based on Artificial Intelligence to reduce costly and time-consuming conventional laboratory testing, validation, and synthetic procedures and accelerate the drug discovery process. We also propose the study of omics data obtained from the innate immune processes of ascidians as an excellent source of innovative bioactive molecules such as antimicrobial, anticancer, and immunomodulatory peptides.

## 2. Bioactive Molecules in Ascidians

Bioactive peptides (BPs) are composed of protein fragments or peptides with beneficial metabolic and physiological functions that promote human health; thus, they are excellent molecules for studying human health and disease and potential therapeutics [43,44]. Most have similar structures, such as <20 amino acid residue lengths, and contain hydrophobic residues [45,46]. Based on their modes of action, different classes of BP are evidenced: anticancer (ACP), antiviral, antimicrobial (AMP), anti-oxidative stress, and immunomodulatory peptides [15,16,17,18,19,47,48].

In the following sections, only AMP and immunomodulatory peptides will be discussed, as they are the only bioactive molecules shown in ascidians.

### 2.1. Bioactive Molecules with Antimicrobial Activity

AMPs are a class of small peptides that exist widely in nature, and they are an important part of the innate immune system of different organisms. AMPs have a broad range of inhibitory effects against bacteria, fungi, parasites, and viruses. The emergence of antibiotic-resistant microorganisms and increasing concerns about the use of antibiotics have resulted in the development of AMPs, which have good application prospects in medicine, food, animal husbandry, agriculture, and aquaculture. Microorganism resistance to antimicrobials is becoming increasingly severe with the abuse of antibiotics in medicine, agriculture, and animal husbandry. The prevalence of vancomycin-resistant enterococcus (VRE) and methicillin-resistant *Staphylococcus aureus* (MRSA) is increasing in clinical medicine, so countermeasures are urgently needed to address these bacterial infections. Research on AMPs is continuously developing, and AMP databases store a considerable amount of data on AMPs. A massive variety of antimicrobials has been extracted from tunicates. They belong to disparate chemical classes, such as polysulfides, alkyl sulfates, terpenes, amino alcohols, spiroketals, alkaloids, furanones, peptides, and others [15]. Some of them are synthesized by symbiotic organisms colonizing the tunic or the internal fluids [49,50].

Most of the known AMPs are produced by ascidian-circulating cells, mainly immunocytes (i.e., cells involved in immune responses) for defense purposes [51,52,53,54,55,56,57,58,59,60] (Table 1).

In *Halocynthia roretzi*, the tetrapeptides halocyamines A and B are produced by cytotoxic morula cells (MCs) [51], and their cytotoxic activity is likely related to their diphenol rings, representing suitable substrates for the enzyme phenoloxidase (PO), which is also stored inside MCs. The enzyme induces oxidative stress by oxidizing phenols to quinones with the consequent production of ROS [64]. The hemocytes of species *H. aurantium* synthesize the peptide dicynthaurin and the cationic peptide halocidin [54]. The native peptide of halocidin has a mass of 3443 Da and comprises two different subunits containing 18 amino acid residues and 15 residues which are linked covalently by a single cystine disulfide bond. Two different monomers were separately synthesized to make three additional isoforms (15-residue homodimer, 18-residue homodimer, heterodimer). Antimicrobial assays performed with synthetic peptides of halocidin confirmed that congeners of the 18-residue monomer were more active than those of the 15-residue monomer MRSA and multidrug-resistant *Pseudomonas aeruginosa*.

Hemocytes from the solitary tunicate *Styela clava* contained a family of four α-helical antimicrobial peptides that were purified, sequenced, and named clavanins A, B, C, and D. Clavanins A–D (histidine-rich, -helix peptides) [49] and clavaspirin are synthesized by *Styela clava* MCs [56]. In lysates of hemocytes of the same species, five cationic antimicrobial peptides, called styelins, were identified and isolated [57,58]. In hemocytes of *Styela plicata*, the octapeptide plicatamide was isolated [59]. In the tunicates *Microcosmus sabatieri* and *Halocynthia papillosa*, antimicrobial activities were detected, and two novel peptides, halocyntin and papillosin, were isolated and characterized. These molecules display antibacterial activity against Gram-positive and Gram-negative bacteria. A combination of Edman degradation and mass spectrometry obtained a complete peptide characterization. The mature molecules of halocyntin and papillosin comprise 26 and 34 amino acid residues, respectively [60].

The enormous quantity of genomic data has become a promising source of putative AMPs due to progress in bioinformatics [65,66,67,68]. In *C. intestinalis*, using genome and expressed sequence tag (EST) data, a putative gene family has been identified exhibiting several structural features typical of AMPs. The synthetic peptide exerted potent antimicrobial activity against various bacteria and against the yeast *Candida albicans*, but it was not cytolytic for mammalian erythrocytes. Using the synthetic peptide as an antigen, specific antibodies were generated, and the natural parent molecule was localized to a compartment of a distinct hemocyte type, the univacuolar refractile granulocytes [53]. Furthermore, a gene family coding for putative AMPs was identified in the EST database of *C. intestinalis* and subsequently identified and cloned from the Northern European *Ciona* subspecies. Molecular analysis revealed that the natural peptide is synthesized and stored in a distinct hemocyte type, the univacuolar non-refractile granulocytes, and that the expression of the gene is markedly upregulated in hemocytes after immune challenge. The peptide proved highly effective against Gram-negative and Gram-positive bacteria, including several human and marine pathogens, as well as the yeast *C. albicans*. Using two different methods, it was demonstrated that the peptide kills Gram-negative and Gram-positive bacteria by permeabilizing their cytoplasmic membranes. Circular Dichroism (CD) spectroscopy revealed that in the presence of liposomes composed of negatively charged phospholipids, the peptide undergoes a conformational change and adopts an alpha-helical structure. Moreover, the peptide was virtually non-cytolytic for mammalian erythrocytes. Hence, this designed AMP may represent a valuable template for developing novel antibiotics [52].

Ci-MAM-A24, a synthetic AMP derived from a peptide precursor from immune cells of *C. intestinalis*, is potently active against representatives of Gram-positive and Gram-negative bacteria by permeabilizing their cytoplasmic membrane. The activity of Ci-MAM-A24 against different bacterial pathogens which frequently cause therapeutic problems was tested. Fedders et al. tested the killing capacity of Ci-MAM-A24 against clinically important anaerobic bacteria as well as multiresistant aerobic strains such as MRSA, VRE, extended-spectrum α-lactamase-producers, and multiple-resistant *Pseudomonas aeruginosa*, and all strains proved to be highly susceptible to Ci-MAM-A24 at low concentrations [69].

Furthermore, an in silico screening method has been developed based on further criteria such as size, amphipathicity, and aggregation propensity, by which 22 potential LCAMP candidates in the *Ciona* genome were computationally predicted. Among these LCAMP candidates, five novel salt-resistant LCAMPs with broad-spectrum antimicrobial activity were experimentally confirmed. This strategy was also successfully applied to the *Xenopus tropicalis* genome, suggesting that this method could apply to the in silico screening of any genome [34].

Finally, Lu et al. [61] investigated the potential sORFs encoding AMPs in *C. intestinalis*, and over 180 peptides deduced from the sORFs were predicted to be AMPs. Among the ten peptides tested, six were found to have significant EST matches, providing strong evidence for gene expression; five were proved to be active against the bacterial strains.

### 2.2. Bioactive Molecules with Immunomodulatory Effects

A recent idea is to use invertebrates as a source of molecules with potential immunoregulatory activities to improve strategies for modulating human immune system responses [65,66]. The innate immune system is composed of many interdependent cell types and mediators. It is one of the most critical natural systems for protection against many harmful bacteria, viruses, parasites, and fungi in human health, and against autoimmune diseases, cancer, allergies, and infections [70,71].

Preliminary studies have shown evidence supporting a complex interaction between the immune system and tumors [72]. Several innate system immunomodulators have been identified; these include cytokines (interleukins, interferons, and chemokines), substances isolated from microorganisms and fungi (lipopolysaccharides; LPS), and substances isolated from plants (polysaccharides and phenolic compounds) [73]. Tumor cells secrete altered protein products that must be recognized as foreign by the immune effector cells such as B, T, natural killer and natural killer T cells, and type I and II interferons, and perforin which are able to destroy tumor cells [74,75]. Therefore, the enhancement of the host immune response is one of the most important methods for inhibiting tumor growth and maintaining cellular homeostasis without harming the host.

The selective modulation of immunity is an emerging concept driven by the tremendous advances in our understanding of this crucial host defense system. Invertebrates have drawn researchers’ interest as potential sources of new bioactive molecules owing to their immunomodulatory activities. An LPS challenge in the ascidian *C. intestinalis* generates the transcript, Ci8 short, with cis-regulatory elements in the 3′ UTR region which is essential for shaping innate immune responses. The derived amino acidic sequence from in silico analysis showed specific binding to human major histocompatibility complex (MHC) class I and class II alleles. The role of Ci8 short peptide (Table 1) was investigated in a more evolved immune system using human peripheral blood mononuclear cells (PBMCs) as an in vitro model. The biological activities of this molecule include the activation of the 70 kDa TCR ζ chain associated protein kinase (ZAP-70) and T cell receptor (TCR) Vβ oligo clonal selection on CD4+ T lymphocytes as well as increased proliferation and IFN-γ secretion. Furthermore, Ci8 short affects CD4+/CD25high-induced regulatory T cells (iTreg) subset selection, which co-expressed the functional markers TGF-β1/latency-associated protein (LAP) and CD39/CD73 [62].

Furthermore, Colombo et al. [63] evaluated the 3D structure of the C8 short-derived *C. robusta* chemo-attractive peptide (CrCP) (Table 1) by homology modeling, which showed that CrCP displayed structural characteristics already reported for a short domain of the vertebrate CRK gene, suggesting its possible involvement in cell migration mechanisms. The biological activity of CrCP was studied in vitro using a primary human dermal cell line. In vitro assays demonstrated that CrCP could induce the motility of HuDe cells in both wound healing and chemo-attractive experiments. Furthermore, CrCP modulates the expression of the matrix metalloproteinase-7 (MMP-7) and E-cadherin genes, and it induces the activation of the NF-κB signaling pathway.

## 3. Bioactive Molecule Identification through Omics Technologies

Classical methods for peptide analysis have relied principally on targeted immunoassays which enable the biochemical purification of bioactive peptides from tissues or cells by identifying fractions with a desired bioactivity [76]. By performing multiple rounds of purification, bioactive peptides could be subsequently identified. This approach discovered many peptides in ascidians, including the isolation of Clavanins in *Styela clava* hemocytes [55] and Halocyamine in the ascidian *Halocynthia roretzi* [51]. During the last decade, technological innovations and the advent of omics data have led to an explosion of biological information. Indeed, different strategies, including transcriptomics and proteomics approaches, such as next-generation sequencing or mass spectrometry, have substituted the classical methods for peptide analysis, producing a considerable amount of biological data. The increasing amount of biological data produced was accompanied by the use of bioinformatics tools to support big data analysis.

Fedders et al.’s initial efforts to integrate genomic data and bioinformatics [53] proceeded through a reverse search for AMPs in *C. robusta*. They used the completed genome project and the substantial amount of EST data available as a screening matrix in association with bioinformatics techniques for the design of synthetic AMPs with in silico tools. Another recent approach to investigating bioactive peptides in *C. robusta* by integrating omics data analysis with bioinformatics focused on the study of the 3D structure of the C8short-derived CrCP, which was evaluated by homology modeling. In vitro studies using a primary human dermal cell line (HuDe) evaluated the biological activity of CrCP. A short domain of the vertebrate CRK gene was identified, suggesting its possible involvement in cell migration mechanisms [75].

Later, Kawada et al. [77] showed how omics studies associated with AI algorithms could contribute to the elucidation of gene expression profiles. These revealed key regulatory genes for *Ciona* follicle growth, maturation, and ovulation, verifying essential and novel molecular mechanisms underlying these biological events with the contribution of machine learning techniques. Furthermore, AI has been employed to solve some of the most challenging issues of bioinformatics, including protein structure prediction, homology searches, multiple alignment and phylogeny construction, genomic sequence analysis, gene finding, and more. Thus, combining omics data with AI and single-cell technologies, Kawada et al. [77] paved the way for investigating in greater detail the nervous, neuroendocrine, and endocrine systems of ascidians and the molecular and functional evolution and diversity of peptidergic regulatory networks throughout chordates. Franchi et al. [78] reported the identification, by mining the *B. schlosseri* transcriptome, of a transcript for a putative styelin-like AMP named botryllin, which is actively transcribed by morula cells (MCs). The synthetic peptide, obtained from in silico translation of the transcript, exerted toxic activity toward bacterial and unicellular yeast cells.

Additional omics data may come from proteome analyses and can also be useful for identifying proteins and peptides with molecule bioactive features, such as AMPs [79]. One of the most widespread proteomic approaches is liquid chromatography coupled with mass spectrometry (LC-MS) [80]. The analysis of spectra obtained by mass spectrometry consists of protein profiling, peptide mapping and identification, and protein quantification. Matos et al. [81], through shotgun proteomics of the ascidian tunic, provided new insights on host–microbe interactions by revealing diverse AMPs. They reported different proteins associated with immune mechanisms of invertebrates, as in the case of the metazoan Down syndrome cell adhesion molecule-like protein, which was detected in *Molgula* sp. samples and related to immune mechanisms mediating phagocytosis and adherence of bacteria [82], or the barrier to autointegration factor (BAF), involved in innate immune response as an inhibitor of exogenous viral DNA replication and involved in host defense response [83], revealing the tunic as a very active tissue in terms of bioactive compound production [81].

A recent study on innate immune response mechanisms to microbial stimuli in a *C. robusta* invertebrate model showed the importance of multi-omics analysis and bioinformatics approaches. Indeed, the intersection of large-scale sequencing or other “-omics” approaches, as the combination of transcriptomic and proteomic data analysis, allows the better investigation of the cellular pathways and biological processes affected by microbial treatments and the investigation of host responses to PAMPs in different physiological conditions and at various stages of maturation of the immune system, thus filling potential gaps concerning expression differences observed in the synthesis of proteins related to mRNA expression [84].

## 4. AI-Based Computational Approaches and Their Role in Drug Discovery

To guide the reader in the following methodological sections, we will briefly introduce the main concepts of AI and its principal subclasses, and then we will focus on AI approaches applied to drug discovery.

AI is a technology-based system that can mimic human intelligence through features such as reasoning, knowledge representation, and solution research. At the same time, it does not threaten to completely replace human physical presence. Moreover, AI can help manage the massive amount of data produced and give decision-making support in clinical and translational research.

Machine learning (ML) is a subclass of AI. Through ML processes, computers can learn without explicitly being programmed. It performs prediction and classification tasks through pattern detection without using defined rules [85]. There are two ML algorithm classes: supervised and unsupervised learning.

In supervised learning, the datasets are designed to train or “supervise” algorithms to classify data or to accurately predict outcomes. Moreover, supervised learning problems can further be divided into “classification” and “regression” tasks: classification assigns test data to specific categories, and regression can predict a continuous numerical output, helping in establishing a relationship among the variables by estimating how one variable affects the other. Another subclass of ML is classification, which is “unsupervised” as it takes references from datasets consisting of input data without labeled responses [85,86,87,88,89,90,91,92].

Due to the massive amount of data produced by NGS techniques, AI has made a significant contribution to data analysis. Also, in bioactive compound analysis, ML algorithms are the basis of many prediction and analysis methods. Deep Learning (DL) algorithms are a subset of ML [93,94,95,96,97,98].

Drug discovery is the process through which new medications against diseases are discovered. It consists of a combination of a wide variety of technologies and expertise aimed at finding potential drugs against specific targets. Typical examples of drug discovery tasks are drug-target prediction [99], bioavailability prediction [100], and de novo drug design [101]. Moreover, there is also the main category of pharmaceutical analysis that groups these examples of drug discovery, involving different subtasks such as toxicity analysis, bioactivity evaluation, and physiochemical property analysis.

**Figure 1:**
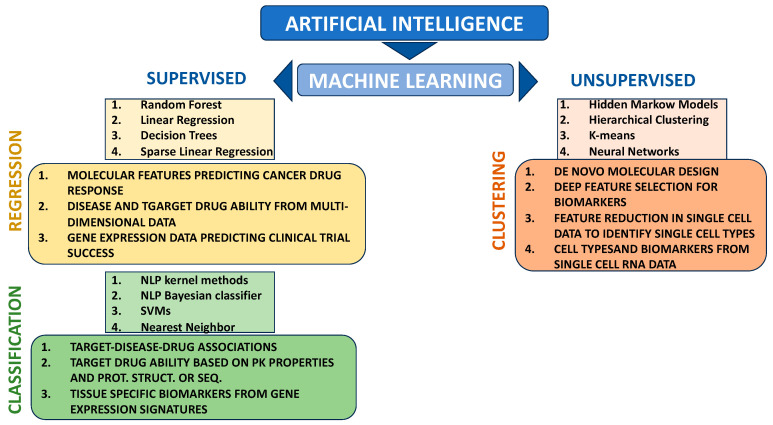
Principal classes and subclasses of AI algorithms and their involvement in drug discovery.

Advancements in AI techniques have revolutionized their applications to this field of research (Figure 1). Indeed, many AI approaches have been developed during the last decade specifically for this aim, allowing the acceleration of the drug discovery process and, at the same time, reducing the high-cost characteristics of conventional methods in terms of money and time [102].

**Figure 2:**
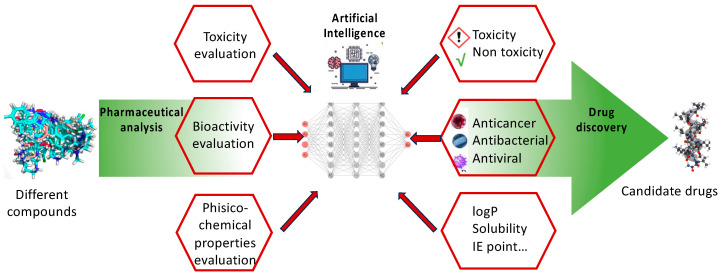
Schematic examples of the main tasks of pharmaceutical analysis and their possible resolution by AI techniques.

Drug toxicity prediction is one of the subtasks of drug discovery, and it allows us to predict how much a molecule could adversely affect humans. The use of AI technologies allows the testing of a specific molecule’s toxicity, avoiding animal tests and high costs (Figure 2) [103,104]. The physico-chemical properties of molecules are another essential feature to assess in drug discovery studies. Their knowledge allows the understanding and modeling of the action of drugs. Among the numerous types of physico-chemical properties are the solubility, molar mass, charge, hydrophobicity, isoelectric point, and percentage of hydrophobic amino acids [105,106]. About 65% of the small molecule drugs are derived from natural products or their derivatives [107]; hence, drug bioactivity assessment has become an active area in drug discovery. AI techniques have been effectively applied to predicting drug bioactivities, such as anticancer, antiviral, and antibacterial activities (Figure 2) [108,109]. Among the different types of bioactive features, antimicrobial properties are gaining much attention as AMPs can be cutting-edge treatments for many infectious disorders. The effectiveness of AMPs against bacteria, fungi, and viruses has persisted for an extended period, making them the best option for addressing the growing problem of antibiotic resistance. Due to their wide-ranging actions, AMPs have become more prominent, particularly in therapeutic applications.

The prediction of AMPs has become difficult for academics due to the explosive increase of AMPs documented in databases. Wet-lab investigations to find antimicrobial peptides are exceedingly costly, time-consuming, and even impossible for some species. Therefore, to choose the optimal AMP candidates before in vitro trials, an efficient computational method must be developed [110]. Bioactive compounds can also assert an essential role as anticancer molecules. Indeed, most of them exert their antiproliferative effects by inhibiting different signaling pathways or intervening in cell-cycle arrest [111]. Various AI-based tools have recently been developed to help solve this aim. Another important subtask of drug discovery is the accurate binding prediction between a major histocompatibility complex (MHC) allele and bioactive peptides, as these last molecules are essential players in the synthesis of personalized cancer vaccines [112]. The immune system struggles to distinguish between a cancerous and a healthy cell. In a patient who has cancer with a particular MHC allele, only those peptides that bind with the MHC allele with high affinity help the immune system recognize the cancerous cells. AI approaches can help predict MHC-II binding, a fast alternative to wet-laboratory investigations, since experiments for MHC class II binding peptide identification are expensive and time consuming.

**Figure 3:**
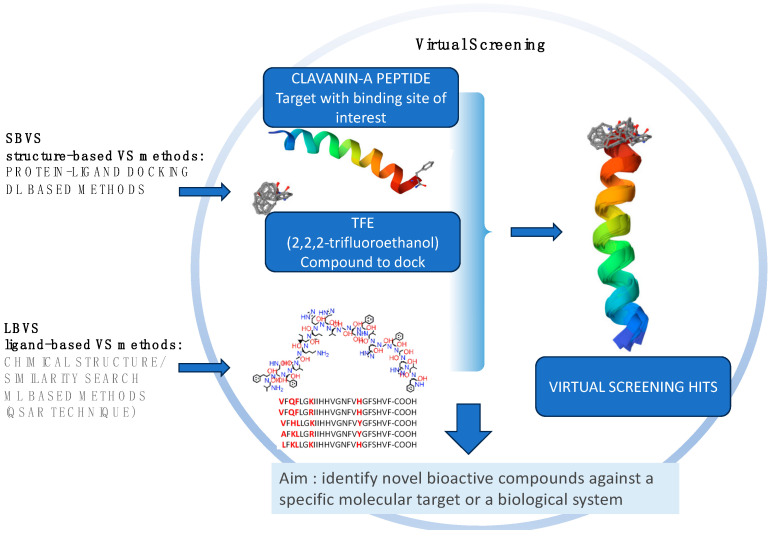
Structure-based and ligand-based VS approaches in protein docking.

Finally, the protein structure is another important aspect to investigate in drug discovery as it allows us to understand the structural interactions, investigating the potential functionality of a protein and acquiring information on important binding domains for target proteins. Indeed, most drug targets are proteins that play essential roles in enzymatic activities, cell signaling, and cell–cell transduction. Although conventional experimental techniques, such as X-ray crystallography, cryogenic electron microscopy, and nuclear magnetic resonance spectroscopy have been proposed to investigate potential protein structures, they are still time-consuming and costly. Therefore, AI technology can help develop novel methods to fill the gap between the number of protein sequences and known protein structures. Virtual Screening (VS) is a computational approach that allows the prediction of the 3D structure of a chemical compound or a potential bioactive molecule against a specific target (Figure 3). It can be divided into ligand-based and structure-based methods. The former is used when very little structural information is available for the target and a set of active ligand molecules is known. Ligand-based methods include pharmacophore modeling and quantitative structure–activity relationship (QSAR) methods. The latter is used to model the interaction between a small molecule with a target protein at the atomic level, thus characterizing the behavior of small molecules in the binding site of target proteins and elucidating fundamental biochemical processes [113].

## 5. AI-Based Web Tools for Bioactive Compound Identification and Analysis

As omics technologies have led to a vast increase in biological knowledge, this raises the necessity of figuring out how to mine helpful knowledge from it, which requires sophisticated data analysis and data-mining methods. To this aim, high-performance computing analysis methods have been developed to support the massive amount of data produced and, at the same time, give decision-making support to clinical and translational research [114,115].

**Figure 4:**
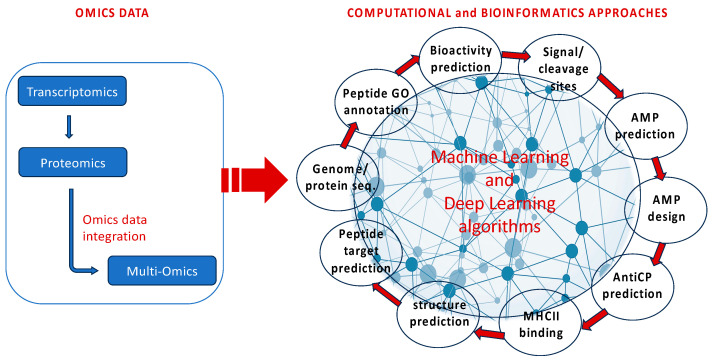
Several types of omics data can be used alone or in an integrated multi-omics approach to be further analyzed by different computational methods based on AI.

By using ML algorithms, computers can learn without explicitly being programmed. It accomplishes prediction and classification tasks through pattern detection without using defined rules. Many bioactive compound analysis and identification methods use ML [116]. Several types of omics data, such as transcriptomics and proteomics, can be used alone or can be integrated into a multi-omics approach to be further analyzed by different computational methods based on AI (Figure 4). Different steps can be completed starting from genome or proteomic sequences to identify and analyze different features of potential bioactive molecules.

Indeed, different tools have been developed based on AI (Table 2):

### 5.1. To predict the Antimicrobial Activity of Peptides

CAMPR3 (http://www.camp3.bicnirrh.res.in/, accessed on 1 October 2023) (Collection of Antimicrobial Peptides) [117,118] allows the expansion and acceleration of AMP family-based studies. It uses information on the conserved sequence signatures captured as patterns and Hidden Markov Models (HMMs) [90]; AmPEPpy is a web tool for predicting AMP sequences using a random forest classifier (https://github.com/tlawrence3/amPEPpy, accessed on 1 October 2023) [119]; InverPrep contains the CALCAMPI algorithm that can calculate the physico-chemical properties (molar mass, charge, hydrophobicity, Boman index, aliphatic index, isoelectric point, and percentage of hydrophobic amino acids) of multiple peptides simultaneously (http://ciencias.medellin.unal.edu.co/gruposdeinvestigacion/prospeccionydisenobiomoleculas/InverPep/public/home_en) accessed on 2 October 2023 [120]; AntiBP2 (http://crdd.osdd.net/raghava/antibp2/, accessed on 2 October 2023) [121] is based on the support vector machine (SVM) algorithm using the composition of peptide sequences. The overall accuracy of the web server is about 92%, and the source of antibacterial peptides can also be predicted; AMPA is a web tool for the prediction of protein antimicrobial regions (http://tcoffee.crg.cat/apps/ampa, accessed on 4 October 2023), and its main application is the fast automatic detection of antimicrobial regions in proteins that can serve as new templates for AMP design. AMPA-derived AI values can be used to classify proteins or domains as antimicrobial or non-antimicrobial automatically and compare different protein sequences in this regard. When used in conjunction with the T-coffee alignment tool, antimicrobial regions can be checked to identify potentially conserved antimicrobial domains [122].

### 5.2. To Identify Peptides with Anticancer Properties

The AntiCP tool (https://webs.iiitd.edu.in/raghava/anticp/, accessed on 4 October 2023) [123] is a web-based prediction server based on machine learning techniques such as SVMs. It can predict every possible single-mutant/analog of a given peptide, and it can also predict their anticancer activity along with all the essential physico-chemical properties like hydrophobicity, charge, isoelectric point, etc. Other anticancer prediction tools using ML or DL models have been developed. Between them is a novel meta-approach which implements a user-friendly webserver for accurately identifying ACPs, which is called MLACP 2.0 [124]. The tool employs 11 different encoding schemes and eight different classifiers, including convolutional neural networks, to create a stable meta-model (https://balalab-skku.org/mlacp2, accessed on 6 October 2023); ACP-MCAM can automatically learn adaptive embedding and the context sequence features of ACP [125]. Chen et al. used the features of amino acid dipeptide composition and pseudo-amino acid composition, combined with an SVM, to construct an ACP prediction algorithm called iACP (http://crdd.osdd.net/raghava/anticp/multi_pep.php, accessed on 6 October 2023) [126].

Other methods can discriminate between ACPs and non-ACPs, and they include ACPP [127], iACP-GAEnsC [128], TargetACP [129], ACPred [130], ACPred-FL [131], ACPred-Fuse [132], ACP-DL [133] and iACP-FSCM [134].

### 5.3. To Predict Peptide Binding with Immune Protein Classes

The automated platform to benchmark peptide–MHC class II binding prediction tools, called the Immune Epitope Database (IEDB), includes different prediction servers, such as NetMHCII 2.3 for T cell epitope prediction, along with other B cell epitope prediction tools (http://tools.iedb.org/main/, accessed on 10 October 2023) [135]. NetMHCII 2.3 (https://services.healthtech.dtu.dk/services/NetMHCII-2.3/, accessed on 10 October 2023) [136,137] uses artificial neuron networks (ANNs) to predict the binding of peptides to HLA-DR, HLA-DQ, HLA-DP, and mouse MHC class II alleles. The user is also guided in their choice of binding strength by strong and weak binding indicated in the output. Other computational methods for the prediction of MHC class II binding include ARB (http://epitope.liai.org:8080/matrix, accessed on 11 October 2023) [138], MHCpred (http://SVRMHC.umn.edu/SVRMHCdb, accessed on 11 October 2023) [139], TEPITOPE (https://github.com/dmnfarrell/epitopepredict, accessed on 11 October 2023) [140], and several others [141].

### 5.4. To Predict AMPs

Prediction tools can be applied to protein sequences from the proteome. A peptide must have many different features to be considered potentially bioactive, such as physico-chemical characteristics, signal peptides, and the location of their cleavage sites. Web tools such as SignalP 5.0 can achieve this aim (https://services.healthtech.dtu.dk/services/SignalP-5.0/, accessed on 13 October 2023) [142]. This web tool can predict the presence of signal peptides or the location of their cleavage sites in proteins from different species through evolution, such as Archaea, Gram-positive Bacteria, Gram-negative Bacteria, and Eukarya. This method uses a combination of several ANNs and HMMs to predict cleavage sites and signal peptides/non-signal peptides. Both ANN and HMM are algorithms belonging to a subset of ML algorithms which are DL models. In ANN, the nodes of the networks are considered to be the neurons of the brain, and the edges connecting the nodes are considered to be “synapses”. DL models have also been applied among AMP predictors. Indeed, a key issue concerning DL models in AMP prediction is the need for samples in the positive class and their ambiguity in the negative class [106]. MultiPep (https://agbg.shinyapps.io/MultiPep/, accessed on 15 October 2023) is a hierarchical DL approach to the multi-label classification of peptide bioactivities [143]. PeptideRanker (http://distilldeep.ucd.ie/PeptideRanker/, accessed on 15 October 2023) [144] predicts peptide bioactivity using bioactivity probability scores. PeptideLocator (http://bioware.ucd.ie/, accessed on 15 October 2023) [145] is based on a BRNN algorithm. Other DL approaches used for different types of peptide analyses are Antimicrobial Peptide Scanner vr.2 (https://www.dveltri.com/ascan/v2/ascan.html, accessed on 16 October 2023) [146] and Deep-AmPEP30 (https://cbbio.cis.um.edu.mo/AxPEP, accessed on 16 October 2023) [147]. Antimicrobial Peptide Scanner vr.2 proposes a neural network model with convolutional and recurrent layers that leverage primary sequence composition to predict AMP properties.

A recent work by Hussain [148] describes an AMP prediction tool called AMP-PFPDeep, which is based on a deep neural network. It improves the accuracy of short antimicrobial peptide prediction using three different sequence encodings and an NN algorithm. The different sequences of the benchmark datasets used in the study were converted into three-channel images comprising information related to the position, frequency, and sum of 12 physiochemical features as the first, second, and third channels, respectively.

AMPGANv2 is a bidirectional conditional generative adversarial network-based approach for rational AMP design (https://gitlab.com/vail-uvm/amp-gan, accessed on 18 October 2023). AMPGAN v2 uses generator–discriminator dynamics to learn data-driven priors and control generation using conditioning variables [149]. The bidirectional component, implemented using a learned encoder to map data samples into the latent space of the generator, aids in the iterative manipulation of candidate peptides.

### 5.5. To Predict Secondary Protein Structure

Web tools that use DL include the PSIPRED Protein Structure Prediction Server from the University College London Bioinformatics Unit (http://bioinf.cs.ucl.ac.uk/psipred/psiform.html, accessed on 20 October 2023) [150], which consists of a two-stage neural network and is based on the position-specific scoring matrices generated by PSI-BLAST. It predicts highly accurate secondary structure predictions.

Another secondary structure prediction method is PSSpred (Protein Secondary Structure prediction) (https://zhanggroup.org/PSSpred/, accessed on 20 October 2023). It also uses PSI-BLAST to collect multiple sequence alignments. Then, amino-acid frequency and log-odds data are used to train the secondary structure, based on the Rumelhart error backpropagation method, and finally, a consensus of seven neural network predictors is used to predict the secondary structure prediction [151]. Among secondary structure prediction tools using DL approaches, there is also the APSSP2: Advanced Protein Secondary Structure Prediction Server [152] http://crdd.osdd.net/raghava/apssp2/ (accessed on 20 October 2023).

### 5.6. To 3D Modeling

The AlphaFold method (https://alphafold.ebi.ac.uk/, accessed on 21 October 2023) [153] is a program that performs predictions of 3D protein structures and is designed as a DL system. IntFOLD (Integrated Fold Recognition) (https://www.reading.ac.uk/bioinf/IntFOLD/, accessed on 21 October 2023) is a fully automated, integrated pipeline for the prediction of 3D structures and functions from amino acid sequences [154]. RaptorX is another DL-based web server predicting both secondary predictions and 3D modeling (raptorx.uchicago.edu, accessed on 24 October 2023) [155]. ESyPred3D is an automated homology modeling program. Alignments are obtained by combining, weighing, and screening the results of several multiple alignment programs. The final three-dimensional structure is built using the MODELLER modeling package [156] (http://www.fundp.ac.be/urbm/bioinfo/esypred/, accessed on 24 October 2023).

### 5.7. Web Tools Employed in VS Techniques

Docking and molecular modeling allow us to know information about molecule orientation and the spatial conformation of the molecule under investigation to infer potential interactions with specific targets as proteins involved in immunity [157]. Some examples of ML-based web servers are OCHEM (https://ochem.eu/home/show.do, accessed on 30 October 2023) [158,159,160,161] and ChemSAR (http://chemsar.scbdd.com, accessed on 30 October 2023) [162], which are both employed in target prediction.

**Table 2:** AI-based tools developed for bioactive peptide analyses.

Drug Discovery Task	Web Tool/Method	AI Algorithm	Ref.	URL
Antimicrobial activity prediction	CAMPR3	HMM (ML)	[118]	http://www.camp3.bicnirrh.res.in/
	amPEPpy	RF (ML)	[119]	https://github.com/tlawrence3/amPEPpy
	InverPrep		[120]	https://InverPep/public/home_en
	AntiBP2	SVM (ML)	[121]	https://webs.iiitd.edu.in/raghava/antibp2/index.html
	AMPA	ML	[122]	http://tcoffee.crg.cat/apps/ampa
	Antimicrobial Peptide Scanner vr.2	ANN (DL)	[146]	https://www.dveltri.com/ascan/v2/ascan.html
	Deep-AmPEP30	CNN (DL)	[147]	https://cbbio.cis.um.edu.mo/AxPEP
	AMP-PFPDeep	DNN (DL)	[148]	https://github.com/WaqarHusain/sAMP-PFPDeep
AMP design	AMPGANv2	generative adversarial network (DL)	[149]	https://gitlab.com/vail-uvm/amp-gan
Anticancer properties	AntiCP	SVM (ML)	[123]	https://webs.iiitd.edu.in/raghava/anticp/
	MLACP 2.0	ANN (DL)	[124]	https://balalab-skku.org/mlacp2
	ACP-MCAM	multi-kernel CNN	[125]	
	iACP	SVM (ML)	[126]	http://lin.uestc.edu.cn/server/iACP
	ACPP	SVM (ML)	[127]	https://github.com/brsaran/ACPP
	iACP-GAEnsC	Genetic Algorithm (ML)	[128]	https://github.com/MLBC-lab/iACP-RF
	TargetACP	SVM (ML)	[129]	
	ACPred	RF+SVM (ML)	[130]	https://github.com/chaninlab/acpred-webserver
	ACPred-FL	SVM (ML)	[131]	http://server.malab.cn/ACPred-FL
	ACPred-Fuse	RF (ML)	[132]	http://server.malab.cn/ACPred-Fuse
	ACP-DL	LSTM (DL)	[133]	https://github.com/haichengyi/ACP-DL
	and iACP-FSCM	Genetic Algorithm	[134]	http://camt.pythonanywhere.com/iACP-FSCM
MHC class II binding prediction	NetMHCII 2.3	ANN (DL)	[135]	https://services.healthtech.dtu.dk/services/NetMHCII-2.3/
	ARB	Average Relative Binding (ARB) matrix	[138]	http://epitope.liai.org:8080/matrix
	MHCpred	PLS-based allele-specific multivariate statistical model	[139]	http://SVRMHC.umn.edu/SVRMHCdb
	TEPITOPEpan	ANN (DL)	[140]	http://www.biokdd.fudan.edu.cn/Service/TEPITOPEpan/
Signal peptides cleavage site location	SignalP 5.0	ANN + HMM (ML + DL)	[142]	https://services.healthtech.dtu.dk/services/SignalP-5.0/
Peptide bioactivity	MultiPep	deep neural network multi-label	[143]	https://agbg.shinyapps.io/MultiPep/
	PeptideRanker	feed-forward neural network (DL)	[144]	http://distilldeep.ucd.ie/PeptideRanker/
	PeptideLocator	BRNN (DL)	[145]	http://bioware.ucd.ie/
Secondary structure prediction	PSIPRED	Two-stage NN (DL)	[150]	http://bioinf.cs.ucl.ac.uk/psipred/psiform.html
	PSSpred	Neural Network (DL)	[151]	https://zhanggroup.org/PSSpred/
	ASPPS2	Neural Network (DL)	[152]	http://crdd.osdd.net/raghava/apssp2/
3D modeling	AlphaPhold	HMM (DL)	[153]	https://alphafold.ebi.ac.uk/
	IntFOLD	Neural Network (DL)	[154]	https://www.reading.ac.uk/bioinf/IntFOLD/
	RaptorX	Neural Network (DL)	[155]	https://raptorx.uchicago.edu/
	ESyPred3D	Neural Network (DL)	[156]	http://www.fundp.ac.be/urbm/bioinfo/esypred/
Virtual Screening	OCHEM	Neural Network	[161]	https://ochem.eu/home/show.do
	ChemSAR	ML	[162]	http://chemsar.scbdd.com

**Figure 5:**
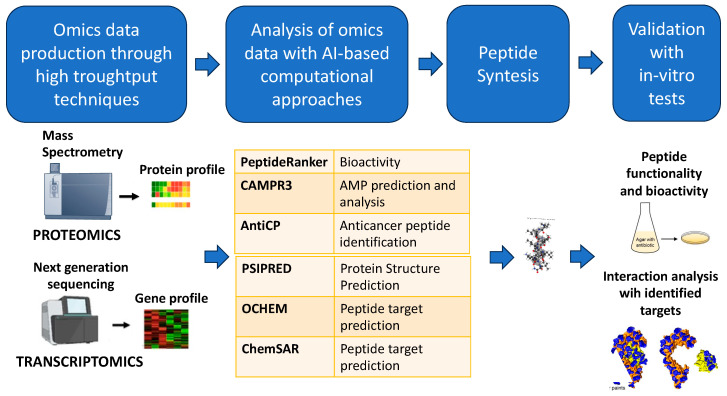
Example of a pipeline used in the study of bioactive molecules with high-throughput omics technologies.

Finally, we show one example of a pipeline used in the study of bioactive molecules with high-throughput omics technologies. Starting from transcriptomics or proteomics of a whole dataset, candidate peptide can be identified and further analyzed through different bioinformatics web tools that allow the evaluation the bioactivity of the putative peptide sequences, the AMP, and the anticancer activity. Moreover, the protein structure of putative peptides and the interaction with target proteins can be predicted. The candidate peptides identified can be then synthetized, and finally, in vitro testing can be used to validate the in silico predictions (Figure 5).

## 6. Conclusions

Drug discovery has always been a complex and time-consuming endeavor that traditionally relies on labor-intensive techniques, such as trial-and-error experimentation and high-throughput screening. However, these methods can be slow, costly, and often yield results with low accuracy. AI techniques such as ML and natural language processing, combined with the experimental production of omics data, offer the potential to accelerate and improve this process by enabling more efficient and accurate analyses of large amounts of data. This combined approach allows researchers to examine a large number of potential drug compounds to identify those with the desired properties. Classic methods can be limited by the availability of suitable test compounds and the difficulty of accurately predicting their pharmacological behavior. Different algorithms based on AI, including supervised and unsupervised learning methods, reinforcement, and evolutionary or rule-based algorithms, can potentially contribute to solving these problems. For instance, the efficacy and toxicity of new drug compounds can be predicted using these approaches with greater accuracy and efficiency than when using traditional methods. Furthermore, AI-based algorithms can also be employed to identify new targets for drug development, such as the specific proteins or genetic pathways involved in diseases. This can expand the scope of drug discovery beyond the limitations of more conventional approaches and may lead to the development of novel and more effective drugs. AI-based methods, on the other hand, can improve the efficiency and accuracy of drug discovery processes and lead to the development of more effective drugs. Furthermore, high-throughput gene sequencing has revolutionized the method used to identify novel molecular targets for drug discovery.

Since there has been increasing attention to finding new solutions for a sustainable supply of bioactive compounds, we would like evidence of the most recent bioinformatics methods to be connected to bioactive compound research. We also propose that a promising source of bioactive molecules, such as ACPs, antiviral, antimicrobial, anti-oxidative stress and immunomodulatory compounds, along with innovative solutions as therapeutic strategies, might come from the study of ascidian innate immunity processes.

Finally, the research’s expected impacts are therefore multiple: from the standardization of a workflow of several techniques that can be replicated by using omics data coming from the study of immune processes of different animal models and different AI based algorithms to the identification of various classes of bioactive molecules that can form the basis for a new groups of drugs which are pharmacologically more efficient and have fewer side effects.

## Figures and Tables

**Table 1:** Bioactive molecules in ascidians from immunocytes: antimicrobial and immunomodulator peptides.

Species	Peptide	Activity	References
*Halocynthia roretzi*	Halocyamines A and B	Antimicrobial	[51]
*Halocynthia aurantium*	Dicynthaurin halocidin	Antimicrobial	[54]
*Halocynthia papillosa*	Halocintin and papillosin	Antimicrobial	[60]
*Styela plicata*	Clavanins A-D	Antimicrobial	[55]
*Ciona intestinalis*	Antimicrobial peptides	Antimicrobial	[34,52,61]
*Ciona robusta*	C8, CrCp	Immunomodulatory	[62,63]
